# Platinum drugs-related safety profile: The latest five-year analysis from FDA adverse event reporting system data

**DOI:** 10.3389/fonc.2022.1012093

**Published:** 2023-01-11

**Authors:** Guowen Feng, Xiaodan Zhou, Jia Chen, Dan Li, Li Chen

**Affiliations:** ^1^ Department of Pharmacy, West China Second University Hospital, Sichuan University, Chengdu, Sichuan, China; ^2^ Key Laboratory of Birth Defects and Related Diseases of Women and Children (Sichuan University), Ministry of Education, Chengdu, Sichuan, China; ^3^ Department of Pharmacy, The People’s Hospital of Langzhong, Langzhong, Sichuan, China; ^4^ University-Town Hospital of Chongqing Medical University, Chongqing, China; ^5^ Department of Pharmacy, Sichuan Provincial People’s Hospital Jinniu Hospital, Chengdu, Sichuan, China; ^6^ The First People’s Hospital of Bijie City, Guizhou, China

**Keywords:** platinum drugs, FAERS, adverse event, date mining, ADE signals

## Abstract

**Background:**

With the widespread application of platinum drugs in antitumor therapy, the incidence of platinum drug adverse events (ADEs) is always severe. This study aimed to explore the adverse event signals of Cisplatin, Carboplatin and Oxaliplatin, three widely used platinum-containing drugs, and to provide a reference for rational individualized clinical drug use.

**Methods:**

The adverse event report data of the three platinum drugs from the first quarter of 2017 to the fourth quarter of 2021 were extracted from the FAERS database, and the data mining and risk factors for the relevant reports were carried out using the reporting odds ratio (ROR) method the proportional reporting ratio (PRR)and the comprehensive criteria (MHRA) method.

**Results:**

A total of 1853 effective adverse event signals were obtained for the three platinum agents, including 558 effective signals for Cisplatin, 896 effective signals for Carboplatin, and 399 effective signals for Oxaliplatin. The signals involve 23 effective different system organs (SOCs). The adverse events of Cisplatin are mainly fixed on blood and lymphatic system diseases, gastrointestinal diseases, systemic diseases and various reactions at the administration site. The adverse events of Carboplatin are mainly focused on blood and lymphatic system diseases, respiratory system, thoracic and mediastinal diseases, while the adverse events of Oxaliplatin are mainly concentrated in respiratory system, thoracic and mediastinal diseases, various nervous system diseases, and gastrointestinal system diseases.

**Conclusion:**

It was found that the main systems involved in common adverse events of platinum drugs are different, and the correlation strength of platinum drugs with the certain adverse events of each system is different.

## Introduction

Since cisplatin was available in the United States in 1978 (1), platinum drugs such as carboplatin (2), nedaplatin, oxaliplatin (3), and lobaplatin have played an extremely important role in the treatment of tumors (4). Adverse drug events (ADEs), such as myelosuppression, nephrotoxicity, allergic reactions, etc., are even life threatening (5, 6). The other one important aspect of platinum resistance is that oxaliplatin is the only platinum drug recommended for platinum resistance current (7).The aim is to choose the appropriate platinum drugs used for chemotherapy, which could help to reduce adverse drug reactions in important target organs and reduce the occurrence of platinum resistance while exerting curative effects (8).

The FDA adverse event reporting system (FAERS), as a global authoritative open-source database for spontaneous reporting of ADEs (9), can make up for shortcomings such as the lag, uncertainty, and incompleteness of the instructions. According to the investigation, the literature was rarely reported based on the FAERS database. The ADEs related to platinum drugs remain unclear in the real world. The authors mine and analyze the ADE signals of platinum drugs in the real world of the last 5 years to explore the differences in ADEs of platinum drugs, to provide a reference for the selection and safe use of platinum drugs and to provide ideas (10) for the development of new drugs.

## Materials and methods

### Data sources and processing

FAERS updates drug ADEs quarterly. This study extracted 20 quarterly data points from the first quarter of 2017 to the fourth quarter of 2021 in the FAERS. To overcome problems with data quality, we manually corrected mistakes in the data entities and deleted duplicates according to FDA’s recommended method, after deleting duplicate reports, screening was performed, and reports with cisplatin, carboplatin, and oxaliplatin as the primary suspected drugs were obtained. ADEs are coded by preferred terms (PTs) in the Medical Dictionary for Regulatory Activities terminology (11, 12). The system organ class (SOC) was also used to classify its ADEs (13).

### Data mining analysis

In pharmacovigilance research, data mining algorithms have been developed for mining adverse reaction signals. The international mainstream signal detection methods include proportional reporting ratio (PRR), reporting odds ratio (ROR) (14, 15), medicine and health products regulatory agency (MHRA), Bayesian confidence propagation neural network (BCPNN) and multiitem gamma pass shrink (MGPS) the empirical Bayes geometric mean (EBGM), etc. The formulas of the ROR method and PRR method in the frequency method are simple, easy to calculate and understand. This study will also use this method for research and analysis. Signal PTs were screened out through the threshold setting, the events in which the lower limit of 95% CI >1 and PTs >3 in the ROR method, and the events with PRR≥2, *χ^2^* ≥4 in which PTs >3 in the MHRA method (12, 16).

## Results

### Basic data of ADE reports

As shown in **Table 1**, the number of reports with cisplatin as the primary suspected drug was 6098, the number of reports with carboplatin as the primary suspected drug was 17640, and the number of reports with oxaliplatin as the primary suspected drug was 12902. Among the patients of known ages, patients over 60 years old accounted for the largest proportion, followed by patients between 18 and 60 years old. Among patients of known gender, adverse events of cisplatin were 51.97% for males and 31.69% for females, carboplatin was 35.52% for males and 45.97% for females, and oxaliplatin was 47.45% for males and 38.14% for females. The top reporting countries are mainly the United States, France, Germany and other European countries.

**Table 1:** Characteristics of 36640 Patients.

Characteristics	Cisplatin	Carboplatin	Oxaliplatin
	N (Proportion)	N (Proportion)	N (Proportion)
age<18y	436 (7.15%)	1040 (5.9%)	34 (0.26%)
18y≤age<60y	2304 (37.78%)	4391 (24.89%)	3993 (30.95%)
age≥60y	2193 (35.96%)	7963 (45.14%)	6717 (52.06%)
Unknown	1165 (19.11%)	4246 (24.07%)	2158 (16.73%)
Male	3169 (51.97%)	6266 (35.52%)	6122 (47.45%)
Female	2201 (36.09%)	8109 (45.97%)	4921 (38.14%)
Unknown	728 (11.94%)	3265 (18.51%)	1859 (14.41%)
Reporting Countries (Top 5)	US (1623, 26.62%)	US (3777,21.41%)	FR (2239,17.35%)
	FR (898, 14.73%)	FR (2808,15.92%)	IT (1797,13.93%)
	DE (507, 8.31%)	IT (1751,9.93%)	US (1562,12.11%)
	IT (494, 8.10%)	DE (1540,8.73%)	NL (91226,9.5%)
	JP (453, 7.43%)	GB (1388,7.87%)	GB (1041,8.07%)

### Comparative analysis

**Table 2** shows that the main ADE symptoms of cisplatin are nausea, febrile neutropenia, and neutropenia; those of carboplatin are dyspnea, neutropenia, and anemia; and those of oxaliplatin are dyspnea, diarrhea, and peripheral neuropathy. The larger the ROR and PRR values, the stronger the signal, and the stronger the correlation between the drug and the adverse event.

**Table 2:** The top 20 ADE signals of Cisplatin, Carboplatin and Oxaliplatin.

Cisplatin			Carboplatin			Oxaliplatin		
PTs	N	95%Cl (ROR)	PTs	N	95%Cl (ROR)	PTs	N	95%Cl (ROR)
Nausea	490	2.17	Dyspnoea	1111	2.32	Dyspnoea	1159	3.42
Febrile neutropenia#	439	23.55	Neutropenia	939	8.65	Diarrhoea	901	2.15
Neutropenia#	387	9.94	Anaemia#	922	6.19	Neuropathy peripheral	799	13.55
Vomiting	372	2.82	Malignant neoplasm progression#	848	8.46	Neutropenia	621	7.62
Acute kidney injury	359	4.85	Febrile neutropenia	839	15.85	Erythema	590	3.63
Pyrexia	323	3.21	Thrombocytopenia	819	9.14	Paraesthesia	558	6.17
Anaemia	297	5.42	Product use in unapproved indication	710	2.21	Hypotension#	453	3.55
Thrombocytopenia	291	8.86	Pancytopenia	692	16.45	Hypersensitivity	442	3.34
Pancytopenia	220	13.86	Pyrexia	673	2.36	Pruritus	431	1.87
Mucosal inflammation	179	21.34	Disease progression	661	8.09	Anaemia	329	2.79
Sepsis	170	4.69	Erythema	551	2.43	Oxygen saturation decreased	313	8.23
Decreased appetite	166	2.15	General physical health deterioration#	491	5.29	General physical health deterioration	293	4.17
Abdominal pain	155	2.17	Sepsis	478	4.88	Hypertension	274	2.09
General physical health deterioration	150	4.3	Hypotension	452	2.56	Malignant neoplasm progression	250	3.08
Hypotension	149	2.27	Acute kidney injury	451	2.06	Loss of consciousness	225	2.87
Leukopenia	141	8.51	Leukopenia	443	10.12	Polyneuropathy	170	19.3
Pulmonary embolism	133	5.3	Abdominal pain	432	2.23	Throat tightness	167	10.05
Malignant neoplasm progression	131	3.26	Neoplasm progression	377	8.43	Dysphonia	161	4.02
Dehydration	128	3.25	Neuropathy peripheral	334	3.76	Chest discomfort	158	2.33
Dysphagia	99	3.26	Flushing	280	3.95	Febrile neutropenia	157	3.47

N, Number of reports; #, represents the maximum ADE signal correlation strength in the top 20 PTs among the three drugs; 95% Cl (ROR), 95% confidence interval (reporting odds ratio).

**Figure 1:**
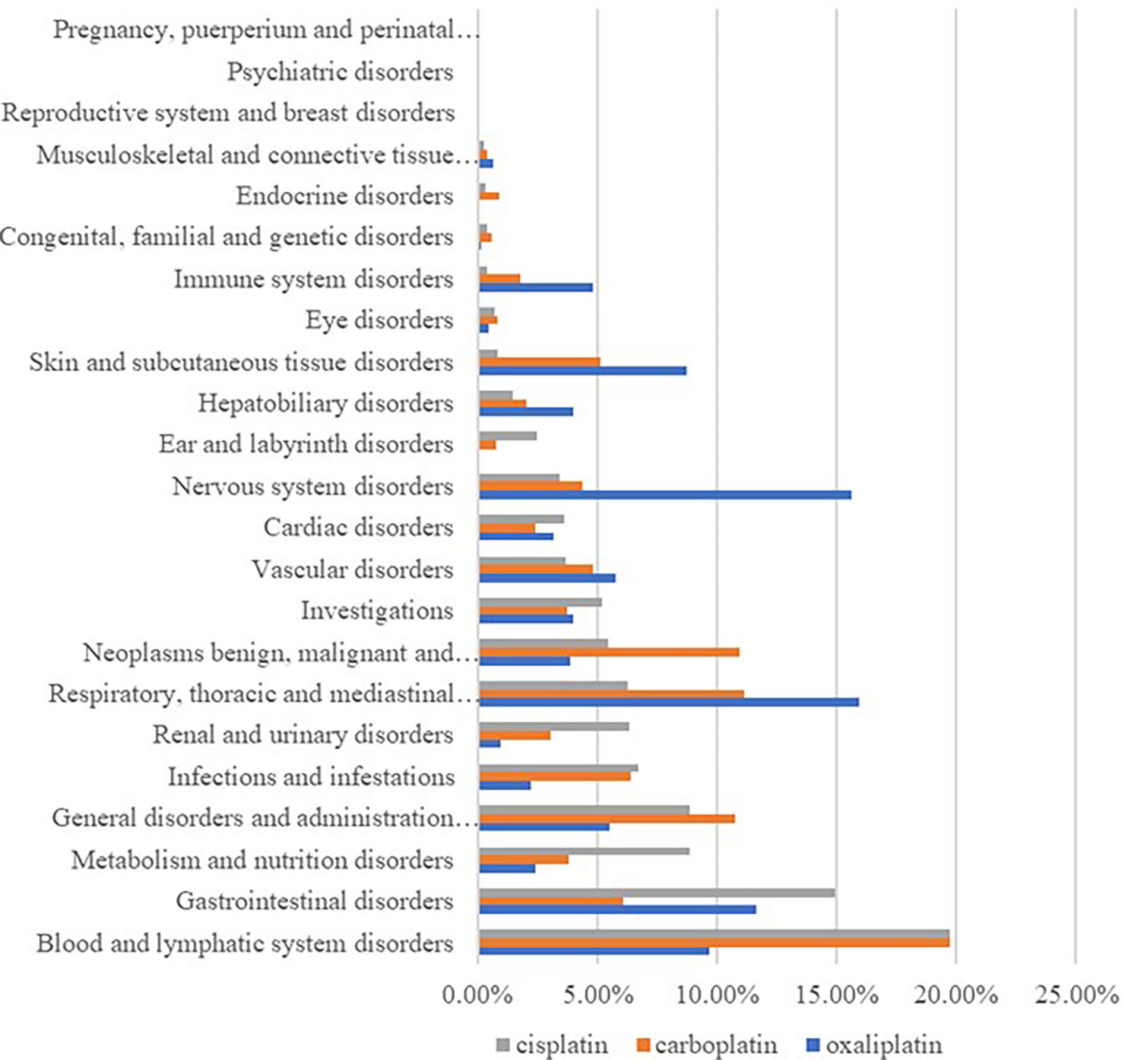
Proportion of reported cases of platinum drugs involving systemic ADEs.

By classifying signaled PTs by SOC, excluding signals unrelated to adverse drug reactions such as product problems, social environment, various injuries, poisoning and operational complications, and various surgical and medical operations, 23 SOCs of all the ADE signals were identified. Among them, cisplatin ADEs involved 21 SOCs, carboplatin ADEs involved 23 SOCs, and oxaliplatin ADEs involved 22 SOCs, and differences in the proportion of reported cases involving platinum-based drugs in systemic ADE are shown in **Figure 1**. Cisplatin ADE reports mainly involve: Blood and lymphatic system disorders, Gastrointestinal disorders, Renal and urinary disorders, Carboplatin ADE reports mainly involve Blood and lymphatic system disorders, Respiratory, thoracic and mediastinal disorders, Oxaliplatin ADE reports mainly involve Nervous system disorders, Respiratory, thoracic and mediastinal disorders, Gastrointestinal disorders.

### Analysis of SOC with prominent signals of platinum drugs

Through data mining, the major adverse events and signal intensity of platinum drugs are quite different.

This result (**Table 3**) suggested that cisplatin had a higher reported rate and the highest correlation with Nephropathy toxic (PRR: 20.52,χ2: 1132.37) and acute kidney injury (PRR: 5.14,χ2: 1206.79). **Table 4** confirms Oxaliplatin is strongly associated with peripheral neuropathy (PRR: 2.74,χ2: 85.33) and paresthesia (PRR: 6.47, χ2: 2570.85). Carboplatin had the highest correlation with polyneuropathy (PRR: 12.31, χ2: 1312.47). **Table 5** confirms that blood and lymphatic system diseases are the most common adverse events caused by platinum drugs, and the incidence rate ranks first among all adverse events. This result suggested that cisplatin had a strong correlation with febrile neutropenia (PRR: 24.17, χ2: 9586.96), which was significantly higher than that of carboplatin and cisplatin. **Table 6** shows that cisplatin has a high reporting rate in nausea and vomiting, and no effective signal was detected by carboplatin and oxaliplatin. There are more reports with carboplatin on abdominal pain and oxaliplatin on diarrhea. Respiratory, thoracic and mediastinal disorder-related adverse events were the most important adverse events of oxaliplatin (**Table 7**). There was a significant difference in the proportion of reported cases among the three drugs. In terms of correlation, cisplatin has a strong correlation with pulmonary embolism (PRR: 6.18, χ2: 577.11), and oxaliplatin has a strong correlation with throat tightness (PRR: 11.59, χ2: 1585.02).

**Table 3:** Kidney and urinary system disorder-related adverse events of Cisplatin, Carboplatin and Oxaliplatin.

	Cisplatin					Carboplatin					Oxaliplatin				
PTs	N	ROR	95%Cl (ROR)	PRR	χ2	N	ROR	95%Cl (ROR)	PRR	χ2	N	ROR	95%Cl (ROR)	PRR	χ2
Acute kidney injury	359	5.40	4.85	5.14	1206.79	451	2.26	2.06	2.23	308.71	–	–	–	–	–
Proteinuria	–	–	–	–	–	88	5.75	4.66	5.73	339.25	–	–	–	–	–
Nephropathy toxic	62	20.72	16.1	20.52	1132.37	84	9.7	7.81	9.66	637.07	18	2.78	1.75	2.78	20.41
Haematuria	–	–	–	–	–	–	–	–	–	–	55	2.48	1.9	2.47	48.11
Renal impairment	55	2.12	1.63	2.11	32.35	–	–	–	–	–	–	–	–	–	–

**Table 4:** Various types of nervous system disorder-related adverse events of Cisplatin, Carboplatin and Oxaliplatin.

	Cisplatin					Carboplatin					Oxaliplatin				
PTs	N	ROR	95%Cl (ROR)	PRR	χ2	N	ROR	95%Cl (ROR)	PRR	χ2	N	ROR	95%Cl (ROR)	PRR	χ2
Neuropathy peripheral	77	2.76	2.22	2.74	85.33	–	–	–	–	–	799	14.57	13.55	13.73	9244.59
Paraesthesia	–	–	–	–	–	–	–	–	–	–	558	6.72	6.17	6.47	2570.85
Loss of consciousness	–	–	–	–	–	–	–	–	–	–	225	3.27	2.87	3.23	347.1
Polyneuropathy	–	–	–	–	–	130	12.4	10.4	12.31	1312.47	–	–	–	–	–
Posterior reversible encephalopathy syndrome	–	–	–	–	–	71	8.26	6.53	8.23	442.33	–	–	–	–	–
Peripheral sensory neuropathy	–	–	–	–	–	69	14.91	11.72	14.86	860.79	61	17.97	13.92	17.89	943.1
Neurotoxicity	29	5.00	3.47	4.98	91.97	67	4.01	3.15	3.99	149.05	–	–	–	–	–
Encephalopathy	–	–	–	–	–	59	3.04	2.35	3.03	79.83	–	–	–	–	–
Ischaemic stroke	–	–	–	–	–	47	2.82	2.11	2.81	54.53	–	–	–	–	–
Depressed level of consciousness	–	–	–	–	–	–	–	–	–	–	56	2.46	1.89	2.45	48

**Table 5:** Blood and lymphatic system disorder-related adverse events of Cisplatin, Carboplatin and Oxaliplatin.

		Cisplatin					Carboplatin					Oxaliplatin				
PTs	N		ROR	95%Cl (ROR)	PRR	χ2	N	ROR	95%Cl (ROR)	PRR	χ2	N	ROR	95%Cl (ROR)	PRR	χ2
Neutropenia	387		11.02	9.94	10.39	3275.14	939	9.25	8.65	8.81	6403.16	621	8.26	7.62	7.91	3721.19
Anaemia	297		6.09	5.42	5.84	1196.9	922	6.62	6.19	6.33	4107.56	329	3.11	2.79	3.06	457.281
Febrile neutropenia	439		25.97	23.55	24.17	9586.96	839	17.01	15.85	16.25	11582.2	157	4.07	3.47	4.03	356.134
Thrombocytopenia	291		9.97	8.86	9.54	2218.13	819	9.81	9.14	9.4	6043.58	–	–	–	–	–
Leukopenia	141		10.06	8.51	9.85	1114.63	443	11.13	10.12	10.88	3878.77	–	–	–	–	–
Haematotoxicity	97		39.38	32.12	38.77	3457.94	170	8.94	7.76	24.04	3547.86	–	–	–	–	–
Bone marrow failure	86		11.09	8.96	10.95	771.29	199	24.27	20.78	8.85	1358.27	–	–	–	–	–

**Table 6:** Gastrointestinal system disorder-related adverse events of Cisplatin, Carboplatin and Oxaliplatin.

	Cisplatin					Carboplatin					Oxaliplatin				
PTs	N	ROR	95%Cl (ROR)	PRR	χ2	N	ROR	95%Cl (ROR)	PRR	χ2	N	ROR	95%Cl (ROR)	PRR	χ2
Abdominal pain	155	2.54	2.17	2.5	141.2	432	2.45	2.23	2.42	360.82	–	–	–	–	–
Diarrhoea	–	–	–	–	–	–	–	–	–	–	901	2.3	2.15	2.21	613.27
Nausea	490	2.38	2.17	2.27	358.58	–	–	–	–	–	–	–	–	–	–
Vomiting	372	3.13	2.82	3	506.07	–	–	–	–	–	–	–	–	–	–
Oesophagitis	–	–	–	–	–	65	8.88	6.94	8.85	443.27	–	–	–	–	–
Colitis	–	–	–	–	–	147	4.66	3.96	4.63	415.02	–	–	–	–	–
Dysphagia	99	3.98	3.26	3.93	216.78	180	2.49	2.15	2.48	157.96	–	–	–	–	–
Ascites	–	–	–	–	–	77	3.37	2.69	3.35	126.4	126	7.61	2.38	7.55	707.38
Gastrointestinal toxicity	–	–	–	–	–	–	–	–	–	–	79	29.91	23.84	29.73	2083.33
Tongue oedema	-	-	-	-	-	-	-	-	-	-	56	24.36	18.63	24.25	1196.93

**Table 7:** Respiratory, thoracic and mediastinal disorder-related adverse events of Cisplatin, Carboplatin and Oxaliplatin.

	Cisplatin					Carboplatin					Oxaliplatin				
PTs	N	ROR	95%Cl (ROR)	PRR	χ2	N	ROR	95%Cl (ROR)	PRR	χ2	N	ROR	95%Cl (ROR)	PRR	χ2
Pulmonary embolism	133	6.30	5.30	6.18	577.11	195	3.16	2.74	3.14	282.77	-	–	–	-	-
Respiratory failure	65	3.25	2.55	3.23	100.03	213	3.71	3.24	3.67	412.2	96	2.26	1.85	2.25	66.95
Interstitial lung disease	33	4.01	2.85	2.37	26.25	164	4.13	3.54	4.1	382.19	140	4.83	4.08	4.79	416.82
Dyspnoea	–	–	–	–	–	1111	2.47	2.32	2.38	904.31	1159	3.63	3.42	3.39	1996.37
Throat tightness	-	-	-	-	-	52	2.61	1.98	2.6	50.97	167	11.73	10.05	11.59	1585.02

Oxaliplatin had a significantly higher incidence of ADEs in immune system disorders than cisplatin and carboplatin (**Table 8**), and oxaliplatin had a strong correlation with type I hypersensitivity (PRR: 49.51, χ2: 4760.50).

**Table 8:** Immune system disorder-related adverse events of Cisplatin, Carboplatin and Oxaliplatin.

	Cisplatin					Carboplatin					Oxaliplatin				
PTs	N	ROR	95%Cl (ROR)	PRR	χ2	N	ROR	95%Cl (ROR)	PRR	χ2	N	ROR	95%Cl (ROR)	PRR	χ2
Hypersensitivity	–	–	–	–	–	–	–	–	–	–	442	3.68	3.34	3.59	827.03
Anaphylactic shock	16	2.45	1.5	2.45	13.67	191	10.41	9.01	10.31	1568.47	124	9.16	7.66	9.08	878.25
Type I hypersensitivity	6	5.33	2.39	5.33	20.99	48	15.22	11.41	15.18	613.34	109	49.92	41.01	49.51	4760.5
Anaphylactoid shock	6	56.98	25.11	56.93	314.57	10	33.85	17.76	33.83	294.27	7	31.63	14.77	31.61	196.43

Cisplatin showed an extremely high correlation in neurosensory hypoacusis (PRR: 416.69, χ2: 3989.40) and mixed deafness (PRR: 169.42, χ2: 439.51). The correlation in Ototoxicity was significantly different for platinum drugs (**Table 9**).

**Table 9:** Ear and labyrinth disorder-related adverse events of Cisplatin, Carboplatin and Oxaliplatin.

	Cisplatin					Carboplatin					Oxaliplatin				
PTs	N	ROR	95%Cl (ROR)	PRR	χ2	N	ROR	95%Cl (ROR)	PRR	χ2	N	ROR	95%Cl (ROR)	PRR	χ2
Neurosensory hypoacusis	13	417.58	221.86	416.69	3989.4	–	–	–	–	–	–	–	–	–	–
Mixed deafness	3	169.51	50.55	169.42	439.51	3	58.48	17.44	58.48	148.29	–	–	–	–	–
Ototoxicity	46	81.19	60.16	80.58	3385.7	79	50.43	39.91	50.21	3394.35	4	3.12	1.17	3.12	5.71

-:No signal detected N: The number of co-occurrences ROR: The reporting odds ratio 95%Cl (ROR): 95% confidence interval (reporting odds ratio) PRR: The proportional reporting ratio χ2: Chi-square test.

## Discussion

As shown in **Table 1**, the number of reports with cisplatin as the primary suspected drug was 6098, the number of reports with carboplatin as the primary suspected drug was 17640, and the number of reports with oxaliplatin as the primary suspected drug was 12902. The top reporting countries are mainly the United States, France, Germany and other European countries. The number of reported cases in Asia, Africa and other populous countries is seriously underestimated for different reasons. Among the patients of known ages, patients over 60 years old accounted for the largest proportion, followed by patients between 18 and 60 years old, which is consistent with the report that tumors occur in the elderly population. Among patients of known sex, carboplatin had a slightly higher number of adverse events reported in female patients than in male patients, and the opposite was true for cisplatin and oxaliplatin, but no gender-related differences were reported. The adverse event signals shown in the data are basically consistent with the instructions, indicating that this study has certain credibility (17).

**Table 3** suggests that cisplatin had the highest correlation with toxic nephropathy and acute kidney injury, followed by carboplatin. Oxaliplatin had the least number of reports on renal and urinary system diseases and the lowest correlation strength, which was consistent with the drug instructions and reports. Therefore, when using these three platinum drugs in patients with primary disease or risk factors for renal disease, cisplatin should be more alert to possible renal injury (8, 18).

The data confirm that neurotoxic is the most report among platinum drugs, with oxaliplatin being more neurotoxic (19). Therefore, more attention should be given to the adverse effects of oxaliplatin on neurological diseases (20), and various neurological diseases during use should be treated in a timely manner (21–23).

Blood and lymphatic system diseases are the most common adverse events of platinum drugs (24); however, effective signals such as thrombocytopenia and leukopenia hematotoxicity have not been detected when using oxaliplatin. Oxaliplatin did not detect an effective signal in febrile neutropenia, Haematotoxicity, Thrombocytopenia, Leukopenia and Bone marrow failure, suggesting that oxaliplatin may be less myelosuppressive than the other two drugs. Oxaliplatin may more suitable for patients with myelosuppression intolerance.

According to the analysis in **Figure 1** and **Table 6**, diseases of the gastrointestinal system are also the main system for the occurrence of adverse events of platinum drugs. Of the three drugs, oxaliplatin was more strongly associated with gastrointestinal toxicity and diarrhea. Cisplatin was more strongly associated with nausea, vomiting, abdominal pain, and dysphagia. As shown in **Table 6**, the association of PTs, such as nausea and vomiting, with carboplatin and oxaliplatin was low (no signal detected), but this does not mean that the incidence of adverse events was low [14]. Carboplatin can be used instead of cisplatin or oxaliplatin when formulating chemotherapy regimens, thereby reducing the risk of gastrointestinal reactions in patients [15, 21, 29].

For ADEs such as dyspnea, throat tightness, skin and subcutaneous tissue system diseases, the incidence and correlation of oxaliplatin were significantly higher than those of carboplatin and cisplatin. Some scholars have proposed initiating cisplatin in patients with proven immediate hypersensitivity to carboplatin or oxaliplatin to ensure safety (25–27). However, during the use of any platinum drug, drug monitoring should be strengthened to prevent allergies and timely symptomatic treatment (28).

Oxaliplatin was more likely to cause allergic reactions (29), and the hypersensitivity and Type I hypersensitivity are particularly evident from the data in this paper. Some scholars have proposed Type II hypersensitivity reactions after oxaliplatin rechallenge can be life threatening (5). A desensitization protocol without premedication may be considered in those patients with a history of oxaliplatin hypersensitivity reactions with avoidance of the cumulative exposure to pretreatment medications (30). Our data suggest that carboplatin and cisplatin may be safer in allergy-prone patients.

A well described and prevalent consequence of cisplatin chemotherapy is ototoxicity, which results from the death of cochlear outer hair cells. Cisplatin-induced ototoxicity is permanent and progressive (31). The data also confirmed the strong correlation of cisplatin in neurosensory hypoacusis. Only 2 effective signals and 11 reports were detected for oxaliplatin, suggesting that the ototoxicity of oxaliplatin is significantly lower than that of cisplatin and carboplatin, and it is safer for children and adolescents (32–34). In many cases oxaliplatin is simply not as effective a drug as cisplatin. The choice has to be a combination of activity and side effects. Such as the effectiveness of cisplatin and carboplatin is higher than oxaliplatin in juvenile trophoblastic tumors and ovarian cancer and they are more widely used. In other words, the effectiveness of the drug is also very important (2022).

This study did not find strong correlation between platinum drugs and cardiac disorders, for all that the cardiotoxicity of platinum-based drugs is of great concern. The mechanisms of cardiotoxicity induced by cisplatin and oxaliplatin include cytotoxicity, oxidative stress and inflammation, cardiomyocyte apoptosis, drug-induced mitochondrial dysfunction and cardiotoxicity (35) and NLRP3 - dependent release of IL - 1 beta induces immune cells, primarily CD4 T cells, to express and release IL - 22 (36).

The well-known Dexrazoxane, A CardioProtective Agent Specific for Anthracy cline Chemotherapy, in addition to Continuous Cardiac monitoring, baseline and regular Electrocardiographic and echocardiographic studies, Carvedilol or Nebivolol, ACE inhibitor, ARB (Sacubitril/Valsartan) or Spironolactone, Statins, Dexrazoxane (37) and Butyric acid can be used to treat or prevent cardiotoxicity caused by chemotherapy drugs (38, 39).

Studies have shown that adiposity is associated with increased cardiometabolic risk after cisplatin-based chemotherapy and higher body mass index were more likely to develop paclitaxel- or oxaliplatin-induced CIPN posttreatment (40, 41), relevant mechanisms need to be further studied.

This study has counted the adverse event outcomes of platinum drugs in the latest five-year. We provide data support for clinical and readers from the perspective of drug safety. The effectiveness and economics of drugs are also important considerations. Which is better to choose oxaliplatin or cisplatin for treatment? The final chemotherapy plan is determined based on many factors, we will investigate in the future work.

Factors such as the level of medical technology, the occupation and professional level of the reporting person, the lack of data such as gender, age, and dosage in the report, the total number of people without drug use, and the randomness of reporting all have a certain impact on the results. Although the ROR method and the MHRA method can reduce the bias caused by the selection of the control group, the obtained results are consistent, and this study increases the accuracy by increasing the threshold of signal detection; it still cannot completely exclude false positive signals. Signals may also be missed. The adverse event signal identified in this study only indicates that there is a statistical correlation between platinum drugs and this signal, but further clinical trials are still needed when choosing drugs in clinical practice.

## Conclusion

In this study, the ROR method and the PRR method were used to perform data mining on the related reports of cisplatin, carboplatin and oxaliplatin. Three drugs have ADE involving the main systems: cisplatin mainly focuses on blood and lymphatic system diseases and gastrointestinal system diseases; carboplatin is mainly concentrated in blood and lymphatic system diseases and respiratory thoracic and mediastinal diseases; oxaliplatin is mainly concentrated in respiratory system, thoracic and mediastinal diseases, various neurological diseases, and gastrointestinal system diseases. Among them, cisplatin has a strong correlation with toxic nephropathy, febrile neutropenia, neurosensory hypoacusis and mixed deafness, carboplatin has a strong correlation with polyneuropathy, and oxaliplatin has a strong correlation with peripheral neuropathy, paresthesia, and type I hypersensitivity. Through data mining and risk factor analysis, it was found that each platinum drug has its own specific ADE, which suggests that oxaliplatin in some diseases may suitable for juvenile patients and is not recommended for patients with allergic constitution. Cisplatin has the greatest nephrotoxicity and ototoxicity. Carboplatin can be used instead of cisplatin or oxaliplatin when formulating chemotherapy regimens, thereby reducing the risk of gastrointestinal reactions in patients. The clinical selection of platinum drugs should be combined with the patient’s relevant medical history and individualized administration and pay more attention to drug effectiveness to reduce the occurrence of adverse events in patients and promote rational clinical drug use.

## Data availability statement

The raw data supporting the conclusions of this article will be made available by the authors, without undue reservation.

## Author contributions

GF, XZ, JC, and DL designed the study. GF, DL, and XZ performed the data analysis. JC managed and checked all the data. All authors contributed equally to the manuscript writing. All authors read, checked, and approved the final manuscript.
